# Fumarate hydratase-deficient uterine leiomyoma: A rare case report and literature review

**DOI:** 10.3389/fonc.2026.1854175

**Published:** 2026-06-24

**Authors:** Qiang Wang, Yanqing Li, Yanshe Shao, Ying Chen, Hui Ren, Xia Ji, Jinying Fu

**Affiliations:** Henan Province Hospital of TCM (The Second Affiliated Hospital of Henan University of Chinese Medicine), Zhengzhou, Henan, China

**Keywords:** 2-succinocysteine, case report, fumarate hydratase, fumarate hydratase-deficient leiomyoma, mutation of fumarate hydrase gene

## Abstract

**Background:**

Uterine leiomyoma is the most common benign tumor in the female reproductive system. Fumarate hydratase (FH)-deficient leiomyoma is a rare subtype characterized by distinctive histopathological and immunophenotypic features and may be associated with hereditary leiomyomatosis and renal cell carcinoma (HLRCC) syndrome.

**Case description:**

A 35-year-old Han Chinese woman, gravida 1 para 1, was admitted in 2021 because of multiple uterine leiomyomas. Pelvic ultrasound demonstrated an enlarged uterus with multiple intramural masses, including a cervical lesion measuring 12.8×10.8×10.3 cm. The patient underwent open abdominal myomectomy. Histopathology showed multiple leiomyomas with focally increased cellularity and occasional mitotic activity. Immunohistochemistry demonstrated CD10 negativity and positivity for SMA, desmin, h-caldesmon, and ER, whereas FH and 2SC staining were not performed at that time. Five months after surgery, multiple intramural masses were detected on ultrasound, but the patient was lost to follow-up for approximately 4 years. In 2025, she presented with irregular vaginal bleeding. Imaging revealed multiple large hypervascular uterine masses with ill-defined margins, heterogeneous echogenicity, and degenerative changes on MRI. The patient reported that both her mother and sister had undergone hysterectomy for uterine fibroids. She subsequently underwent total hysterectomy with bilateral salpingo-oophorectomy. Pathological examination demonstrated multiple intramural leiomyomas with degeneration and focal infarction. Immunohistochemistry revealed loss of FH expression and diffuse strong 2SC positivity,confirming FH-deficient uterine leiomyoma. Abdominal CT showed no renal abnormality. FH genetic testing, genetic counseling, and lifelong renal and dermatologic surveillance were recommended, but the patient declined genetic testing. At 6-month follow-up, she remained asymptomatic without imaging evidence of recurrence.

**Conclusion:**

FH-deficient uterine leiomyoma should be suspected in young women presenting with large, multiple, recurrent, hypervascular leiomyomas and familial clustering of uterine fibroids. Early FH/2SC immunohistochemical screening, genetic counseling, and long-term renal surveillance are important for identifying suspected HLRCC risk and guiding individualized management. Even when germline FH testing is declined, patients with FH-deficient uterine leiomyoma should not be managed as routine leiomyoma cases, but should receive counseling regarding suspected HLRCC risk, dermatologic assessment, and lifelong renal surveillance.

## 1 Introduction

Uterine leiomyomas are the most common benign tumours of the female reproductive system, with a prevalence of up to 70% among women (1). Among these, FH-deficient uterine leiomyomas represent a rare subtype, accounting for approximately 1%-2% of all uterine leiomyomas (2, 3). These tumours exhibit distinctive histomorphological features and molecular alterations associated with germline or somatic mutations in the FH gene, and may serve as a sentinel manifestation of hereditary leiomyomatosis and renal cell carcinoma (HLRCC) syndrome. Patients typically present at a younger age, with fibroids often being multiple, large in size, and prone to recurrence (4). However, the clinical manifestations overlap with those of conventional uterine leiomyomas, which can delay recognition if FH/2SC immunohistochemical screening is not performed. The clinical value of recognizing this subtype is not limited to the uterine lesion itself. It also lies in identifying patients who may require syndromic risk assessment, renal surveillance, dermatologic examination, genetic counseling, and family risk assessment. The present case is reported because it illustrates several practical issues frequently encountered in clinical practice:early-onset and large multiple leiomyomas, early postoperative detection of recurrent or residual lesions within 5 months after myomectomy, progressive tumor enlargement after loss to follow-up, familial clustering of uterine fibroids, delayed FH/2SC testing, and refusal of germline FH testing despite pathological evidence of FH deficiency. This report therefore highlights not only the diagnostic features of FH-deficient uterine leiomyoma but also the management challenges of a suspected HLRCC/FH tumor predisposition risk when molecular confirmation is unavailable.

## 2 Case description

In January 2021, a 35-year-old Han Chinese gravida 1 para 1 woman with one previous vaginal delivery was admitted because of multiple uterine leiomyomas. Before admission, she had developed abnormal vaginal bleeding, dark-red discharge, bleeding volume approximately twice her usual menstrual flow, blood clots, and mild lower abdominal pain, with moderate anemia. On gynecological examination, the uterus was anteverted, enlarged to approximately a 20-week-size uterus, hard, irregular on the surface, mobile, and mildly tender. Pelvic ultrasound revealed an enlarged uterus with abnormal morphology and multiple uterine masses. A fundal lesion measured approximately 7.8×6.0 cm on 2D ultrasound, and a cervical lesion measured 12.8×10.8×10.3 cm. Punctate and linear blood flow signals were visible on Doppler imaging (**Figures 1A, B**). She underwent open abdominal myomectomy. Postoperative pathology revealed multiple uterine leiomyomas with focally increased cellularity and occasional mitotic figures. Immunohistochemistry showed CD10 negativity and positivity for SMA, desmin, h-caldesmon, and ER. The Ki-67 index was approximately 1%, PHH3 showed 1 positive mitosis per 10 high-power fields, and CD34 highlighted vascular endothelial cells. FH and 2SC immunohistochemical staining were not performed on the initial myomectomy specimen at that time. In June 2021, routine postoperative surveillance ultrasound was performed 5 months after myomectomy. Transvaginal ultrasound revealed an enlarged uterus with multiple solid, slightly hypoechoic masses in the anterior and posterior uterine walls. The largest lesion was located in the posterior uterine wall and measured approximately 3.7×3.0 cm on 2D ultrasound. Minimal internal blood flow signals were observed (**Figures 1C, D**). This early postoperative finding suggested rapid recurrence or residual growth, although the distinction between true recurrence and residual microscopic disease could not be determined retrospectively. The patient did not continue regular follow-up thereafter.

**Figure 1:**
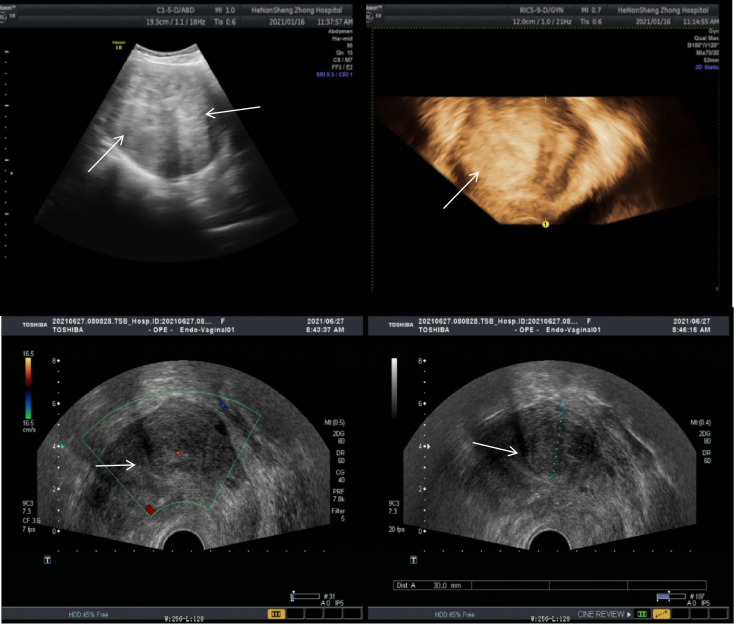
Transvaginal ultrasound images before and after the first surgery. **(A, B)** Preoperative transvaginal grayscale and color Doppler ultrasound images in January 2021 show a large, solid, slightly hypoechoic mass involving the uterine body and cervix. The lesion demonstrates heterogeneous echogenicity and distortion of the normal uterine architecture. Solid arrows indicate architectural distortion of the uterine wall. **(C, D)** Routine postoperative surveillance ultrasound in June 2021 shows multiple solid hypoechoic masses in the uterine wall. The largest lesion is located in the posterior uterine wall. Arrowheads indicate recurrent or residual myometrial lesions.

In September 2025, the patient was admitted again because of recurrent uterine fibroids with progressive enlargement over approximately 4 years. She presented with 8 days of irregular vaginal bleeding, abdominal pain, low back pain, a pelvic bearing-down sensation, dizziness, fatigue, chest tightness, poor appetite and disturbed sleep. On gynecological examination, the uterus was anteverted, enlarged to more than a 24-week-size uterus, with the fundus reaching the umbilical level, extending to the right midaxillary line, and showing poor mobility. A firm uterine mass measuring approximately 12.0×11.0×10.0 cm was palpable in the uterine body. Pelvic ultrasound revealed an abnormally enlarged uterus with distorted morphology, along with multiple uterine masses. The largest mass was located in the uterine body and measured approximately 13.0×11.3×8.0 cm. A cervical mass measured approximately 3.1×2.2 cm on 2D ultrasound. Both lesions showed poorly defined borders, heterogeneous internal echogenicity, and linear blood flow signals at the periphery and within the lesions. An echogenic mass with irregular internal echoes was visible in the uterine cavity,measuring approximately 11.0×2.1 cm on 2D ultrasound, with well-defined borders and no detectable blood flow. A tortuous tubular echo was observed in the left adnexal region, measuring approximately 2.0 cm at the widest diameter, without internal blood flow signals. No obvious abnormality was found in the right adnexal region (**Figure 2**). Ultrasound examinations of the abdomen and urinary system showed no significant abnormalities. Serum tumor markers, including CA125, CA19-9, CEA, AFP, inhibin, LDH, and SCC, were within normal ranges. Pelvic MRI was performed in addition to ultrasound. MRI revealed multiple uterine masses. The largest lesion measured approximately 12.0×10.4×9.7 cm, was located mainly in the submucosal region, and showed degenerative changes (**Figure 3**). Given the abnormal vaginal bleeding, intracavitary echogenic lesion and atypical imaging features, concomitant endometrial pathology and malignant potential were considered preoperatively. However, based on a comprehensive evaluation that took into account the patient’s age, clinical presentation, and preoperative ultrasound/imaging findings, the diagnosis was more likely to be a benign lesion, with a relatively low probability of malignant endometrial disease. Therefore, no further endometrial biopsy was performed preoperatively.

**Figure 2:**
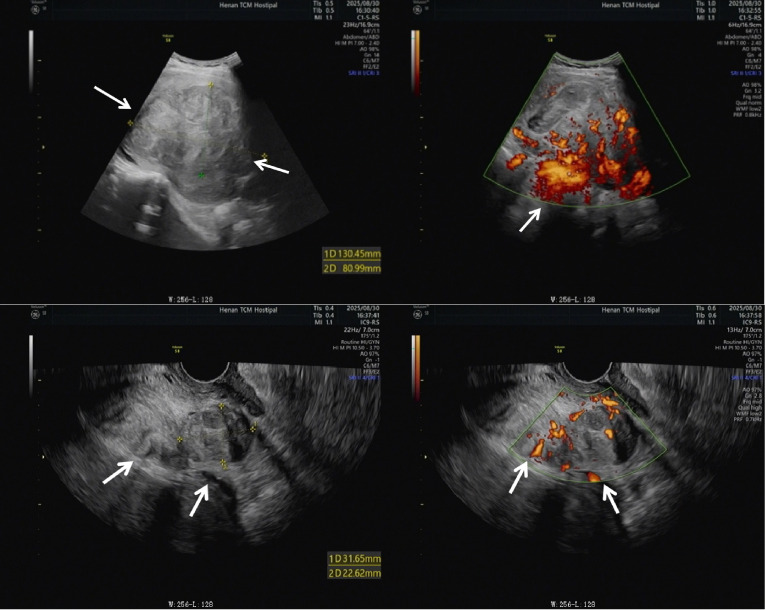
Transvaginal ultrasound images before the second surgery. Transvaginal grayscale and color Doppler ultrasound images show an enlarged and distorted uterus with multiple solid hypoechoic masses. The largest uterine body mass shows poorly defined borders, heterogeneous internal echogenicity, and peripheral and intralesional linear vascular signals. A separate intracavitary echogenic lesion is visible without detectable blood flow.

**Figure 3:**
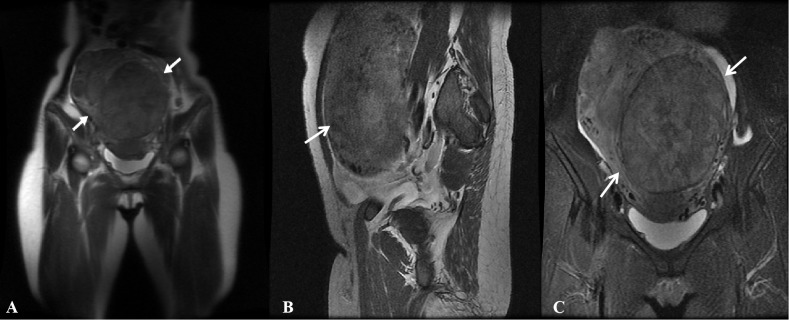
Pelvic MRI before the second surgery. **(A)** Coronal, **(B)** Sagittal, and **(C)** Axial MRI images demonstrate multiple nodular and mass-like lesions within the uterus. The largest lesion is heterogeneous and shows mixed signal intensity with degenerative changes. Contrast-enhanced imaging shows marked heterogeneous enhancement with patchy non-enhancing areas, while smaller lesions show relatively uniform enhancement. Most lesions have indistinct borders with the surrounding normal myometrium.

The patient had a family history of uterine fibroids. Her mother and sister had undergone total hysterectomy due to uterine fibroids. After discussion with her family,the patient requested definitive surgery. Following informed consent, she underwent total hysterectomy with bilateral salpingo-oophorectomy. Intraoperatively, the uterine body measured 15.5×15.0×9.5 cm. Grossly,the tumor tissue was grayish white and firm, and the largest component measured 11.0×10.0×8.5 cm. Pathological examination revealed multiple intramural uterine leiomyomas with degeneration, consistent with FH-deficient uterine leiomyomas, with focal infarction in some areas. Histologically, the tumor cells showed oval to spindle-shaped nuclei, focal atypia, intracytoplasmic eosinophilic granules and staghorn-like vessels. The eosinophilic nucleoli and perinuclear halos were not prominent. The mitotic count was 0–2 per 10 high-power fields. Immunohistochemistry showed CD10 negativity, positivity for SMA and desmin, loss of FH expression, strong 2SC positivity, a Ki-67 index of approximately 5%, and focal p16 positivity (**Figure 4**). Based on the histomorphological features and immunohistochemical profile, the final diagnosis was FH-deficient uterine leiomyoma. Further FH gene testing and genetic counseling were recommended. Renal imaging was also recommended to exclude renal neoplastic involvement associated with hereditary leiomyomatosis and renal cell carcinoma syndrome. Abdominal CT showed no renal abnormality. Because the patient was asymptomatic after surgery and was concerned about the implications and cost of genetic testing, she declined FH genetic testing and chose regular follow-up. The patient recovered well after surgery. At the 6-month follow-up,she remained asymptomatic and pelvic ultrasound showed no abnormal findings. A timeline of the case is shown in **Figure 5**.

**Figure 4:**
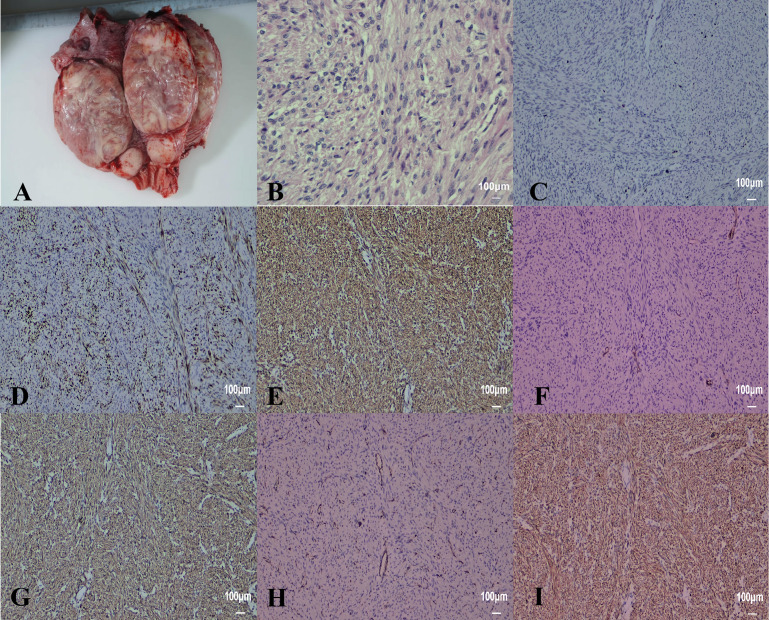
Gross, histomorphological and immunohistochemical findings of the hysterectomy specimen. **(A)** Gross image of the hysterectomy specimen photographed on a clean background. The cut surface shows a grayish-white, firm, whorled tumor. **(B)** Hematoxylin and eosin staining shows spindle tumor cells with oval to rod-shaped nuclei, focal nuclear atypia and staghorn-like vessels. The eosinophilic nucleoli and perinuclear halos were not prominent. Mitotic activity was assessed at 400× magnification and was 0–2 mitoses per 10 high-power fields. (HE, ×400). **(C)** Ki-67 immunostaining, proliferation index approximately 5%, 100×. **(D)** p16 immunostaining, focally positive, 100×. **(E)** Desmin immunostaining, positive, 100×. **(F)** CD10 immunostaining, negative, 100×. **(G)** SMA immunostaining, positive, 100× **(H)** FH immunostaining,lost in tumor cells with retained internal positive control, 100×. **(I)** 2SC immunostaining, strongly positive, 100×.

**Figure 5:**
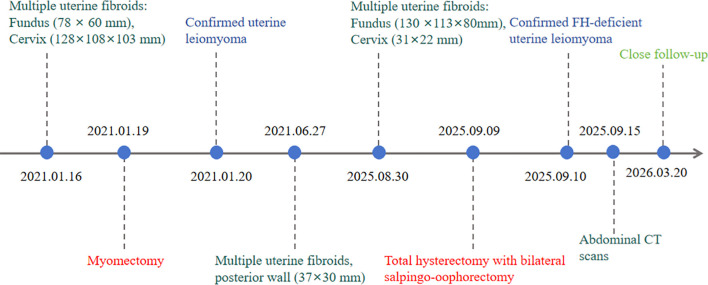
A timeline with relevant data from the episode of case.

## 3 Discussion

FH-deficient uterine leiomyoma is a rare but clinically important subtype of uterine leiomyoma. Its incidence is higher in younger patients, accounting for approximately 2.6% of uterine leiomyoma cases in patients younger than 40 years and approximately 4.6% in patients younger than 30 years (5, 6). The age of onset in the present patient is consistent with the reported tendency for FH-deficient leiomyomas to occur in relatively young women. FH-deficient leiomyomas are driven by germline or somatic alterations in the FH gene. Popp et al.reported that most uterine leiomyomas associated with FH deficiency are sporadic, with approximately 2.7%-13.9% of patients carrying germline FH mutations (7, 8). Germline FH mutations cause hereditary leiomyomatosis and renal cell carcinoma syndrome, which is characterized by cutaneous leiomyomas, early-onset uterine leiomyomas, and aggressive renal cell carcinoma (9). McHenry et al.further showed that HLRCC-associated uterine leiomyomas are often multiple, large, and early-onset, leading to surgical treatment at a younger age than in patients with sporadic leiomyomas (10). In contrast, somatic FH mutations are more common in sporadic FH-deficient leiomyomas. Ahvenainen et al. reported that sporadic FH-deficient uterine leiomyomas may also show unusual clinical behavior, including metastatic potential in rare cases (11). Therefore, accurate recognition of FH-deficient leiomyoma is clinically important because it may identify patients who require genetic counseling and renal surveillance.

FH is a tumor suppressor gene located on chromosome 1q42.3–q43. It encodes fumarate hydratase, a key enzyme in the tricarboxylic acid cycle that catalyzes the reversible hydration of fumarate to malate (12). The pathogenesis of FH-deficient leiomyoma is not fully understood. Proposed mechanisms include dysregulation of the tricarboxylic acid cycle, fumarate accumulation, pseudohypoxic signaling, abnormal protein succination, oxidative stress, altered iron homeostasis, and impaired homologous recombination repair (13). These molecular changes provide a biological basis for the distinct histological and immunohistochemical features of FH-deficient tumors. Although the clinical manifestations of FH-deficient uterine leiomyomas, such as abnormal uterine bleeding and uterine enlargement, overlap with those of conventional leiomyomas, this subtype has distinctive pathological features (14, 15). In the present case, staghorn-like vessels, nuclear atypia, and intracytoplasmic eosinophilic granules were observed, whereas perinuclear halos and alveolar-like edema were not prominent. Immunohistochemistry showed loss of FH expression and strong 2SC positivity. The combination of morphology, FH loss, and 2SCpositivity supported the diagnosis of FH-deficient uterine leiomyoma (16, 17).

This case is clinically noteworthy for several reasons. First, the patient was young and had multiple large uterine leiomyomas. Second, recurrence was detected only 5 months after myomectomy during routine surveillance, and the tumors continued to enlarge over the following years. Third, she had a family history of uterine fibroids, as both her mother and sister underwent hysterectomy for fibroids. These features are consistent with previous observations that FH-deficient uterine leiomyomas, particularly those associated with germline FH mutations, may occur earlier, be multifocal, and cluster within families. Liu et al.reported that patients with FH-deficient uterine leiomyoma carrying germline FH mutations had earlier onset, more frequent multifocality, higher rates of surgical intervention, and stronger familial clustering, than patients without germline FH mutations (18). However, FH/2SC immunohistochemical staining was not performed during the initial myomectomy in this case. As a result, FH deficiency and the potential hereditary cancer risk were not recognized at the first operation. This reflects a practical challenge in routine pathological practice: FH-deficient leiomyoma may be overlooked if the tumor is interpreted as a conventional leiomyoma and FH/2SC staining is not included. For young patients, especially those younger than 45 years, and for patients with large, multiple, recurrent, atypical, or familial leiomyomas, FH/2SC immunohistochemical screening should be considered.

Once FH-deficient uterine leiomyoma is identified, the key clinical issue is not only local uterine management but also hereditary risk assessment. Clinicians should document family history, attempt to construct a three-generation pedigree, and specifically ask about renal tumors, early-onset kidney cancer, cutaneous leiomyomas, and early-onset uterine fibroids. Genetic counseling and FH germline testing are recommended to distinguish sporadic tumors from HLRCC-associated disease. For patients with confirmed or strongly suspected HLRCC, renal surveillance is essential because renal cell carcinoma associated with HLRCC may be aggressive and can occur at a young age. In this case, abdominal CT showed no renal lesion, but the patient declined FH genetic testing because she was asymptomatic and concerned about the implications and cost of testing. She agreed to regular follow-up. This highlights the need for better patient education regarding hereditary cancer risk and the value of genetic assessment, while also highlighting a practical challenge in clinical practice. In addition, the patient underwent surgical treatment for severe symptomatic uterine leiomyomas before the age of 40. Her mother and sister developed severe symptomatic uterine leiomyomas before the age of 40 and underwent surgical treatment. Although a complete three-generation family tree was not constructed, no cases of renal cell carcinoma, renal tumors, or pathologically confirmed cutaneous leiomyomas were identified among her first- and second-degree relatives. Therefore, in the absence of FH mutation testing, this patient is classified as having FH-deficient uterine leiomyomas with suspected but unconfirmed HLRCC risk, rather than clinically confirmed HLRCC. Based on this, she should be advised to undergo dermatologic assessment, lifelong annual renal MRI surveillance, repeated genetic counseling, and family-history reassessment, regardless of the patient’s current asymptomatic status. The paper should serve as a guide for how clinicians should handle these cases when molecular confirmation is absent.

## 4 Conclusion

FH-deficient uterine leiomyoma is a rare subtype of uterine leiomyoma and may serve as an important marker for hereditary leiomyomatosis and renal cell carcinoma syndrome. Diagnosis relies on careful histomorphological evaluation, FH/2SC immunohistochemical screening, and, when indicated, FH genetic testing. In young patients with multiple, large, recurrent, atypical, or familial leiomyomas, clinicians and pathologists should consider FH-deficient leiomyoma in the differential diagnosis. Surgical treatment remains the main approach for symptomatic or recurrent uterine disease, but recognition of FH deficiency is particularly important because it can guide genetic counseling, family risk assessment, and renal surveillance. Rather than preventing malignant transformation of the leiomyoma itself, timely diagnosis primarily helps identify patients at risk for hereditary renal cell carcinoma and supports appropriate long-term monitoring.

## Data Availability

The original contributions presented in the study are included in the article/Supplementary Material. Further inquiries can be directed to the corresponding author.
